# Evaluation of Antioxidative Mechanisms In Vitro and Triterpenes Composition of Extracts from Silver Birch (*Betula pendula* Roth) and Black Birch (*Betula obscura* Kotula) Barks by FT-IR and HPLC-PDA

**DOI:** 10.3390/molecules26154633

**Published:** 2021-07-30

**Authors:** Aleksandra Ostapiuk, Łukasz Kurach, Maciej Strzemski, Jacek Kurzepa, Anna Hordyjewska

**Affiliations:** 1 Department of Medical Chemistry, Medical University of Lublin, 4A Chodźki Str., 20-093 Lublin, Poland; a.ostapiuk98@gmail.com (A.O.); jacek.kurzepa@umlub.pl (J.K.); annahordyjewska@umlub.pl (A.H.); 2 Independent Laboratory of Behavioral Studies, Medical University of Lublin, 4A Chodźki Str., 20-093 Lublin, Poland; 3 Department of Analytical Chemistry, Medical University of Lublin, 4A Chodźki Str., 20-093 Lublin, Poland; maciej.strzemski@poczta.onet.pl

**Keywords:** *Betula pendula* Roth, *Betula obscura* Kotula, silver birch, black birch, pentacyclic triterpenes

## Abstract

Silver birch, *Betula pendula* Roth, is one of the most common trees in Europe. Due to its content of many biologically active substances, it has long been used in medicine and cosmetics, unlike the rare black birch, *Betula obscura* Kotula. The aim of the study was therefore to compare the antioxidant properties of extracts from the inner and outer bark layers of both birch trees towards the L929 line treated with acetaldehyde. Based on the lactate dehydrogenase test and the MTT test, 10 and 25% concentrations of extracts were selected for the antioxidant evaluation. All extracts at tested concentrations reduced the production of hydrogen peroxide, superoxide anion radical, and 25% extract decreased malonic aldehyde formation in acetaldehyde-treated cells. The chemical composition of bark extracts was accessed by IR and HPLC-PDA methods and surprisingly, revealed a high content of betulin and lupeol in the inner bark extract of *B. obscura*. Furthermore, IR analysis revealed differences in the chemical composition of the outer bark between black and silver birch extracts, indicating that black birch may be a valuable source of numerous biologically active substances. Further experiments are required to evaluate their potential against neuroinflammation, cancer, viral infections, as well as their usefulness in cosmetology.

## 1. Introduction

Over 140 species of trees of the genus *Betula* are known worldwide [1]. Within Europe, three naturally occurring species of high commercial significance are particularly noteworthy: silver birch (*Betula pendula* Roth), white birch (*Betula alba*) and *B. pubescens* [2]. The extracts and the compounds present in them have been commercialized on a small scale and constitute the basis of dietary supplements, cosmetic care products, or biocides. Many birch species are characterized by antibacterial activity—this has been proven experimentally in *B. utilis*, *B. pendula*, or *B. papyrifera* [3]. In turn, birch sap, extracted in early spring from the trunk, was used as an aid in the treatment of kidney and urinary tract diseases, skin diseases and also rheumatism or gout [1]. Various phytochemical studies have shown that *B. pendula* extracts contain mainly terpenes, polyphenols, including flavonoids, saponins, and sterols [4]. The most important compounds obtained in the bark extraction process are terpenes and their derivatives. Such compounds include lupeol, erythrodiol, oleanic acid, betulin, and its derivatives, betulinic acid, and betulinic aldehyde [5]. 

**Figure 1:**
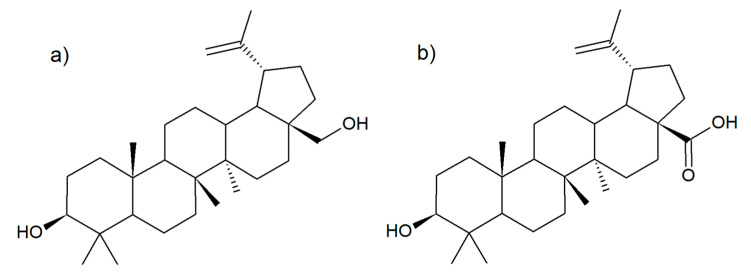
Chemical structure of betulin (**a**) and betulinic acid (**b**).

Betulin (lup-20(29)-ene-3β,28-diol) (Figure 1a) is a pentacyclic lupine-type compound. Although this compound can easily be extracted from more than two hundred species of plants, the richest source are the birch family of trees, in particular, white birch (*B. alba*) and silver birch (*B. pendula*) [6]. Betulin is also responsible for the characteristic white color of the silver birch bark, filling the inside of periderm cells [7]. A derivative of betulin, formed during its oxidation, is betulinic acid (3β-hydroxy-lup-20(29)-en-28-oic acid) (Figure 1b). This compound is also present in the outer bark of white birch, although in a much smaller amount [8]. 

The applications of products from *B. pendula* extracts are very widespread, due to the wide pharmacological and physiological effects of the compounds they contain. Betulin regulates the production and distribution of melanin in the skin by inhibiting the tyrosinase enzyme. This enzyme is responsible for converting tyrosine into melanin dye. Thus, *B. pendula* is reported to be used in the prevention and care of skin with melanin synthesis disorders [9]. Moreover, betulinic acid has been tested for anticancer properties, and the pioneer study in this regard was the work of Pisha et al., which showed the considerable cytotoxic effects of betulinic acid on melanoma cells [10]. Research conducted for over two decades since the pioneering discovery in 1995 has revealed many other valuable properties of betulin and betulinic acid, including antiviral (especially in relation to HIV), anti-inflammatory, hepatoprotective or antifungal activity [11]. However, the antioxidant activity of betulin and betulinic acid, whether in the form of pure substances or the form of plant extracts, still remains one of their most important properties. 

The oxidation process is based on the transfer of electrons between atoms and is an important part of metabolism and ATP synthesis. The sudden interruption of the electron flow results in the transfer of single, unpaired electrons and thus the generation of free radicals [12]. Reactive oxygen species (ROS) are formed in many cellular processes, among others as by-products of mitochondrial and chloroplastic electron transport chains, and in response to a number of stress factors. [13]. In low concentrations, ROS act as messenger molecules, contributing to controlling the development and response to environmental factors [14]. They are also produced in the body as part of the primary immune response [15]. In a healthy cell, there is a balance between pro-oxidation and antioxidation. However, affecting this balance by overproducing ROS or reducing the level of antioxidants causes free radicals to rise rapidly. This condition is called oxidative stress [16]. 

**Figure 2:**
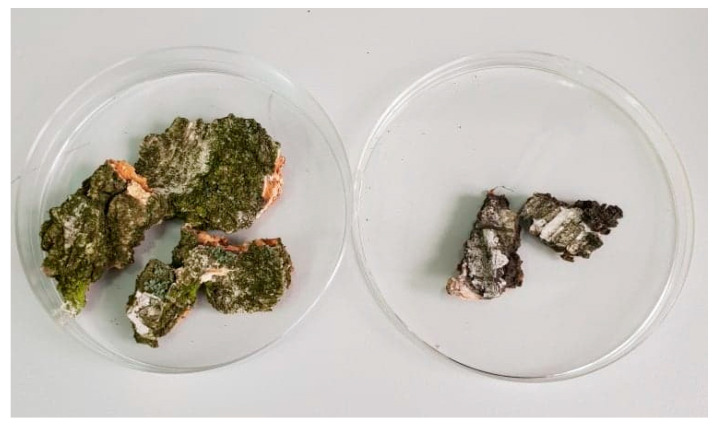
*Betula obscura* Kotula (left) and *Betula pendula* Roth (right) outer barks.

Unlike *Betula pendula*, the black birch (*Betula obscura* Kotula) is a rare birch species, predominantly occupying forest areas of central Europe, mainly occurring in Poland, Slovakia, Ukraine, and the Czech Republic. In Poland, it is found mainly in the south and its distribution and variability have been described by Hrynkiewicz-Sudnik as a subendemic species [17]. A discrepancy arises in the recognition of *Betula obscura* Kot. as a separate species. Like most of the studies on this topic, the first official description clearly separates the black birch from other plants of its kind, giving it the name of a new species. A similar view is also represented by Stecki et al. [18], the authors of probably the most accurate descriptions of the morphology and anatomy of *Betula obscura*. Notwithstanding, many other studies consider black birch only as a form or subspecies of *B. pendula* [19]. The most significant difference between those two species is probably the absence of the thin-walled cells with betulin crystals in the cork cambium of *B. obscura*. This results in the differences in the bark color—it is described as cherry gray, not white (Figure 2). Nowadays, according to The Plant List, *B. obscura* Kotula is recognized as a distinct species [20].

Since many pharmaceutical formulations based on natural extracts showed promising biological activity, and there are hardly any scientific reports on the biological activity of *B. obscura*, we decided to investigate the properties of *B. pendula* Roth and *B. obscura* Kotula bark extracts as potential antioxidants in an in vitro model of the L929 cell line (murine fibroblast cells) treated with acetaldehyde. Cell viability was analyzed by the MTT and LDH assays. The level of oxidative stress parameters, including the production of O_2_^•−^, H_2_O_2_, and MDA assay, was assessed. In order to confirm the diversity of species and show the chemical composition pattern, the absorption spectra of the inner and outer bark of both birches obtained by infrared spectroscopy were analyzed. Furthermore, qualitative analysis of triterpenes, betulinic acid, betulin, lupeol was performed.

## 2. Results

### 2.1. Chemical Profile of Barks’ Extracts by Fourier Transform Infrared Spectroscopy

Fourier transform infrared (FT-IR) spectroscopy provides structural information on the molecular features of a large range of compounds. The identification of the major chemical groups of the examined compounds is usually based on the “fingerprint” region (950–1200 cm^−1^) [21]. 

From FT-IR investigations of bark extracts (Figures S1–S4 in Supplementary Materials) it can be said that there are no differences between the inner barks as both spectra are similar. The chemical pattern of the outer bark extracts posed several different signals, reflecting the diverse composition. The most characteristic common bands for both bark extracts are ~2929, ~2868 cm^−1^ corresponded to stretching ν(CHx), ~1448 cm^−1^ to bending δ(CH_x_), ~1028 cm^−1^ to stretching ν(C-O) and deformation δ(CH) + ρ(CH_3,_ CH_2_), ~880 cm^−1^ to wagging ω(H-C-H) vibrations. Significant differences were the absence of a band around ~3342 cm^−1^ (ν(OH)), and the presence of medium 1259 cm^−1^ and strong 800 cm^−1^ bands in *B. obscura* outer bark extract. The spectra of pure betulin and betulinic acid (Figures S5 and S6) were rather similar, but two characteristic bands were used to distinguish them, namely ~1684 cm^−1^ corresponding to ν(C=O) and bands in the range 1032–1006 cm^−1^ which reflect the triterpene composition, as betulin produces a strong band at 1008 cm^−1^, whereas betulinic acid exhibits a medium signal at 1032 cm^−1^. The comparison of the obtained IR bands of the outer bark extracts and the reference standards of betulin and betulinic acid are listed in Table 1. Assignment of bands was done according to experimental and theoretical literature data [22,23].

### 2.2. Quantification of Pentacyclic Triterpenes by HPLC

**Figure 3:**
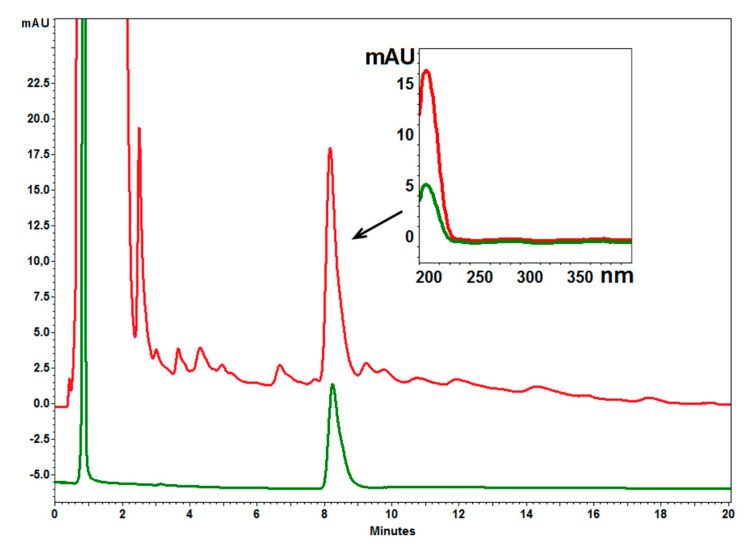
Example chromatograms of birch bark extract (red line) and reference compound—lupeol (green line). Over the chromatograms the spectrum of identified and reference compound. RP18e Chromolith, acetonitrile–water (95:5 *v*/*v*), flow rate 2 mL/min, at 25 °C.

**Figure 4:**
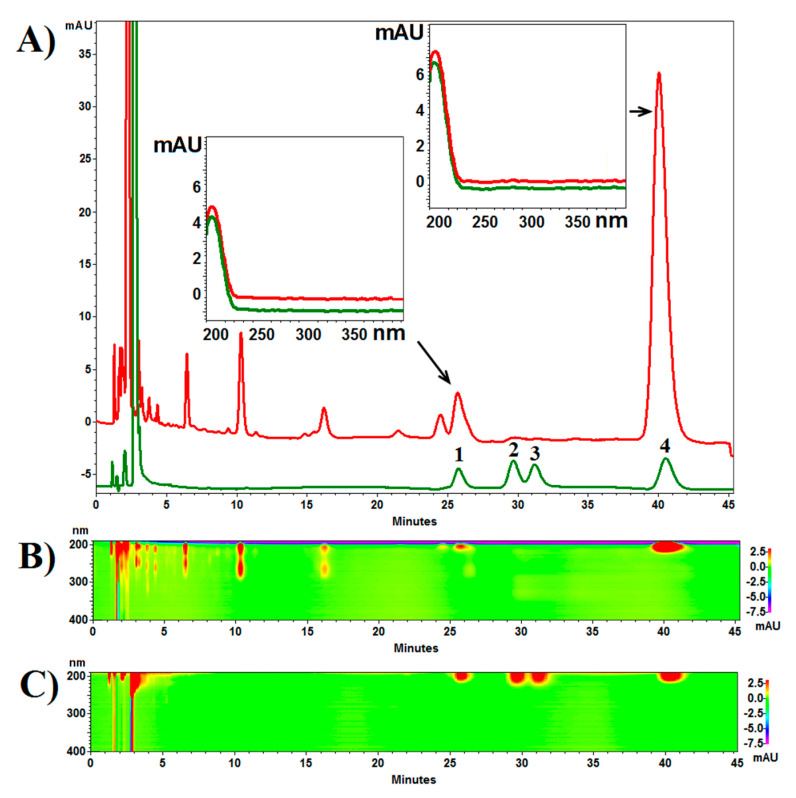
(**A**) Example chromatograms of birch bark extract (red line) and reference compounds (green line): 1 (betulinic acid), 2 (oleanolic acid), 3 (ursolic acid), 4 (betulin). Over the chromatograms the spectra of identified and reference compounds. (**B**,**C**); The spectro-chromatograms of extract and reference compounds, respectively. RP18e LiChro-spher, acetonitrile–water—phosphoric acid aqueous solution at concentration of 1% (75:25:0.5, *v*/*v*/*v*), flow rate 1.0 mL/min at 10 °C.

The pentacyclic triterpene composition of the dried extracts from both *B. pendula* and *B. obscura* was analyzed by a reversed-phase liquid chromatography (RP-HPLC-PDA). Chromatograms are presented in Figure 3 and Figure 4. 

Analysis revealed a higher content of betulin and lupeol in the inner bark extract of *B. obscura* than in *B. pendula*, while the opposite was in the outer bark extract. The lupeol content in the outer bark extract was similar. A similar concentration of betulinic acid was also found in all four extracts (Table 2).

### 2.3. Extract Cytotoxicity Analysis

The toxicity of the *B. pendula* and *B. obscura* barks extracts towards the L929 cell line was measured by two mechanism-independent assays—MTT and LDH. MTT reflects mitochondrial enzyme activity whereas the lactate dehydrogenase assay indicates the percentage of membrane-compromised cells undergoing death and thus releasing the LDH directly into the surrounding area [24]. 

As a result of a 24 h exposure to the extracts of 2–100% concentration, it was observed that the viability of the L929 cells remained inversely proportional to the increase in the concentration of extracts (Table 3 and Table 4). After 24 and 48 h, no visible alteration in cell viability incubated with the 2% of extract was demonstrated in MTT, and with 2% and 5% in the LDH assay, whilst a statistically significant decrease in viability was demonstrated for *B. pendula* inner bark of 10% for 24 h (*p* < 0.5) and for 48 h (*p* < 0.0001) by MTT, 25% (*p* < 0.001) and 50% (*p* < 0.0001) for the 24 h and 48 h treatments, respectively, by LDH. In the case of outer bark, a significant statistical decrease was observed for 10% (*p* < 0.0001 and 5% (*p* < 0.01) by MTT after 24 h and 48 h incubation, 25% (*p* < 0.001) and 50% (*p* < 0.0001) by LDH for 24 h and 48 h treatments, respectively.

For *B. obscura* inner bark, MTT assay revealed a statistically significant decrease in regard to a viability of 10% (*p* < 0.0001) and 5% (*p* < 0.01) and for the 24 h and 48 h treatments, respectively. Whereas the LDH revealed a statistically significant decrease in regard to viability of 10% (*p* < 0.01) and 25% (*p* < 0.001) for the 24 h and 48 h treatments, respectively. For outer bark, MTT showed a decrease for 10% (*p* < 0.0001) and 5% (*p* < 0.0001) for 24 h and 48 h, respectively, whereas, the LDH assay 25% for 24 h (*p* < 0.05) and 48h (*p* < 0.0001).

### 2.4. Antioxidant Mechanism of Bark Extracts

In order to evaluate the antioxidant potential of *B. pendula* and *B. obscura* bark extracts against biological ROS in the cellular environment, H_2_O_2_, O_2_^•−^ production, and MDA concentrations were accessed. Acetaldehyde was used as ROS inducer (positive control). Two concentrations of the extracts were selected—10 and 25%. Concentrations higher than 50% were excluded, as they may affect the results by their impact on cell viability. Meanwhile, lower concentrations were eliminated due to too low, potentially biologically irrelevant, concentrations.

#### 2.4.1. Hydrogen Peroxide (H_2_O_2_) and Superoxide Anion Radical (O_2_^•−^) Concentration Assessment Prove the Antioxidant Activity of Both *B. pendula* and *B. obscura* Bark Extracts

**Figure 5:**
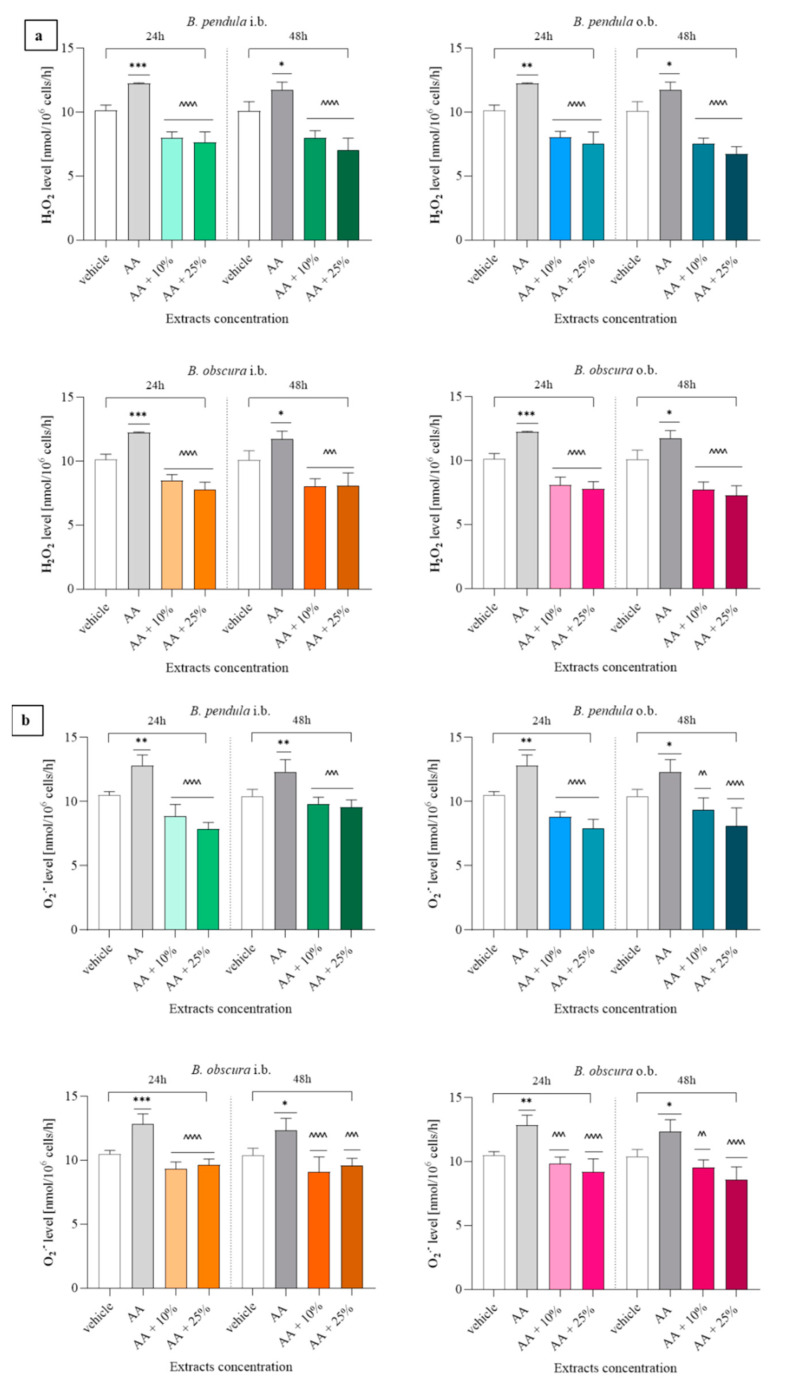
Evaluation of hydrogen peroxide (**a**) and superoxide anion radical (**b**) release from 24 h and 48 h incubation of L929 cell line with 10% and 25% extracts. Data presented as the means ± SD; * *p* < 0.05, ** *p* < 0.01, *** *p* < 0.0001, vs. vehicle group; ^^^^ *p* < 0.01, ^^^^^ *p* < 0.001, ^^^^^^ *p* < 0.0001 vs. AA group; Tukey’s test. Abbreviations: i.b., inner bark; o.b., outer bark; AA, acetaldehyde (positive control).

The results demonstrated that *B. pendula* inner and outer bark, *B. obscura* outer bark extracts regardless of the incubation time (24 h and 48 h), extract concentrations (10 and 25%), exhibited inhibitive properties towards the release of H_2_O_2_ when added to the acetaldehyde-stimulated cells (*p* < 0.0001) (Figure 5a). *Betula obscura* inner bark extract after a 24 h incubation decreased the release of H_2_O_2_ (*p* < 0.0001), and after 48 h (*p* < 0.001) in both tested concentrations. Examination of O_2_^•−^ release reveals that, as for H_2_O_2_, the synthesis of superoxide anion radical was lower in cells cultured with 10 and 25% extracts of *B. pendula* inner and outer bark, *B. obscura* inner bark (*p* < 0.0001), *B. obscura* outer bark 10% (*p* < 0.001), and 25% (*p* < 0.0001) extracts (Figure 5b). After a 48 h incubation, O_2_^•−^ release was attenuated for cells treated with *B. pendula* inner bark 10 and 25% extract (*p* < 0.001), *B. pendula* outer bark 10 (*p* < 0.01) and 25% (*p* < 0.0001) extract, *B. obscura* inner bark 10% (*p* < 0.0001) and 25% (*p* < 0.001), *B. obscura* outer bark 10 (*p* < 0.01) and 25% (*p* < 0.0001) extract. 

#### 2.4.2. Malonic Dialdehyde (MDA) Concentration Assessment Indicates Ongoing Antioxidant Properties of All Birch Bark Extracts

**Figure 6:**
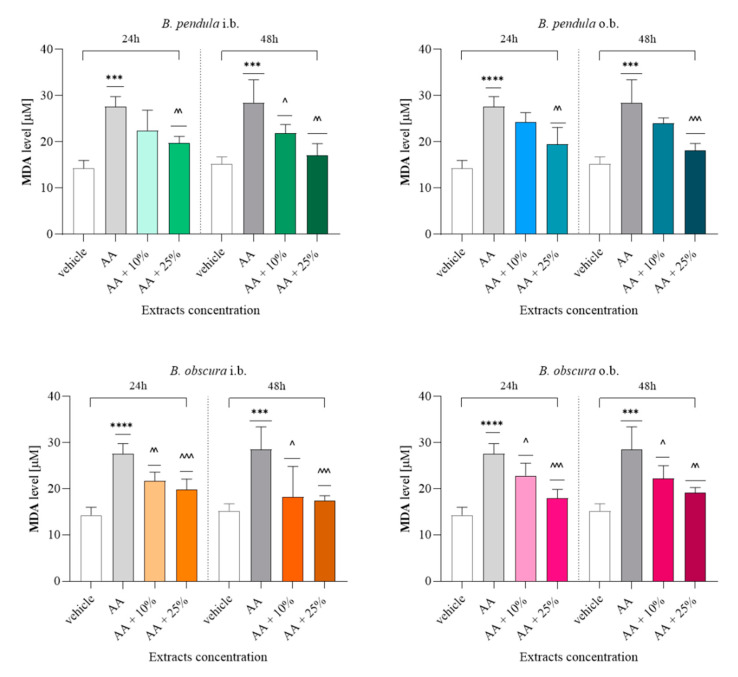
Assessment of malonic dialdehyde (MDA) release from 24 h and 48 h incubation of L929 cell line with 10% and 25% extracts. Data presented as the means ± SD; *** *p* < 0.001, **** *p* < 0.0001 vs. vehicle group; ^^^ *p* < 0.05, ^^^^ *p* < 0.01, ^^^^^ *p* < 0.001 vs. AA group; Tukey’s test. Abbreviations: i.b., inner bark; o.b., outer bark; AA, acetaldehyde (positive control).

The MDA assay showed that the release of malonic dialdehyde was lower in cells cultured with 10% extracts of *B. obscura* inner bark (*p* < 0.01), outer bark (*p* < 0.05) after 24 h. (Figure 6). It has been observed that the 25% extract reduces lipid peroxidation to a greater extent than the 10% extract in case of *B. pendula* outer bark after 24 h (*p* < 0.01), and after 48 h (*p* < 0.001), *B. obscura* outer bark after 24 h (*p* < 0.001), and 48 h (*p* < 0.01). The 25% inner bark extracts of *B. pendula* and *B. obscura* regardless of the incubation time also showed an antioxidant effect (*p* < 0.01 and *p* < 0.001, respectively).

## 3. Discussion

In the era of growing demand for natural sources of medications, cosmetics, or generally, biologically useful substances, more emphasis is put on examining the impact of the substances in nature on the physiological and pathological processes occurring in the human body. 

One of the plants the research focused on was *B. pendula* Roth (also known as silver birch), a plant from Betulaceae family, abundantly found in Europe. During advanced phytochemical studies, it has been proven that the silver birch bark extracts are a rich source of triterpenes, betulin in particular [25]. Betulin is located in the form of crystalline clusters in large, thin-walled cells appearing in the spring and, by filling periderm cells, is responsible for the white color of the bark [7]. In much smaller, yet still significant amounts, a betulin derivative, betulinic acid, is also present in the *B. pendula* bark extracts [26]. In recent years, numerous studies have been conducted that have revealed many valuable properties of both betulin and betulinic acid: antiviral (against influenza, anti-HIV), antiallergic, anti-inflammatory, hepatoprotective, antiallergic, or antituberculosis [27]. 

In the presented study, an attempt has been made to elucidate the antioxidant potential of a relatively unknown birch species, which is *B. obscura* Kotula, also known as black birch. It is a barely endemic species, known to occur mainly in central Europe, predominantly in Poland. Hitherto, there was not sufficient source material to determine the so far known biological applications of *B. obscura* in a similar range to *B. pendula*. It might have seemed that due to the lack of the white color of the black birch bark, the species lacks at least some of the pentacyclic compounds, including betulin, characteristic for the silver birch. Therefore, it could be hypothesized that *B. obscura* may not possess as many of the biological properties of *B. pendula*. Thus, as this assumption required further experimental support and no available data could be obtained from the literature, this study was designed. Yet, this pioneering approach of including a species relatively unknown to science also finds it impossible to compare the obtained results with those previously presented in the literature. In none of the studies published so far have the antioxidant activities of *B. pendula* and *B. obscura* been compared. Interestingly, hardly any existing sources also directly focus on the antioxidant properties of *B. pendula* or particularly on its bark extracts. Of the betulin-rich birch species, articles usually focus on *B. alba*, the white birch. We found this lack of literature intriguing, as *B. pendula* is a species abundant in betulin, the antioxidant properties of which have already been widely described. 

The work of Mashentseva et al. [1] compared the antioxidant properties of various vegetative parts of *B. pendula* and clearly presented the bark as an organ with the greatest antioxidant potential. Though, it was not specified which part of the bark was used for the study. Antioxidant properties were evaluated by several methods, namely DDPH, ABTS, FRAP, additionally the total phenolic and flavonoid content was measured in dry extract. Although the tests used to assess the antioxidant properties differ from those used in our study, the final conclusions overlap, confirming the antioxidant activity of the silver birch bark. Moreover, it can be conjectured that due to the presence of betulin and betulinic acid in the extracts of white-barked birches, these compounds will at least partly affect their biological activity. Based on numerous studies, including Szuster-Ciesielska et al. [28], it can be concluded that the in vitro properties of betulin include antioxidant potential. Rzeski et al. [29] also provided numerous evidence for the anticancer effects of betulin, both in vitro and in vivo. One of the speculated pathways of betulin activity is the prevention of ROS-caused apoptosis. A similar conclusion, however—due to the action of betulinic acid—is drawn by many, including Szuster-Ciesielska et al., Zheng et al., and Yi et al. [28,30,31]. Ultimately, Jafari Hajati et al. [32] presented results combining the inhibition effect of free radical scavenging activity of *B. pendula* with the presence of betulin and betulinic acid in its bark callus.

Considering the research oriented on elucidating the antioxidant properties of naturally-derived substances, it is so far the only study comparing a plant with recognized and documented antioxidant potential—*B. pendula*—and *B. obscura*, barely unknown to literature. The first aim of this study was to characterize the antiproliferative properties of the bark extracts. The largest decrease in cell viability, compared to the control cells, was demonstrated in cultures exposed to birch bark extracts at a concentration of 50% and higher. No differences were observed in the results depending on the used species of birch, the type of bark used, or the incubation time of the cells with the extracts. Our study indicates that both extracts used to assess oxidative stress parameters—at both 10 and 25%, regardless of birch species or bark type—showed antioxidant activity in the acetaldehyde treated cells to which they were added. The synthesis of both hydrogen peroxide and the superoxide anion radical was reduced in cells incubated with extracts, compared to positive control cells. Using the MDA assay, it was observed that both extracts used to reduce the level of lipid peroxidation in cells treated with acetaldehyde, and the 25% extract reduced lipid peroxidation to a greater extent than the 10% extract. No significant differences were dependent on the time of incubation, on the species or type of birch bark used.

The obtained results are promising and confirm the antioxidant properties of all four tested extracts. We have shown the first evidence of the antioxidant properties of *B. obscura* that could contribute to its inclusion in the group of birches with antioxidant properties. In view of the antioxidant theory of aging, or the impact of free radicals in neurodegeneration or cancer, discovering new sources of antioxidants is currently highly desirable. As for many years, natural medicine has benefited from the use of birch bark, we provide a scientific basis to explain at least part of their properties. We have also shown that despite differences in exposure to environmental factors, both external and internal bark show comparable antioxidant ability.

The antioxidant activity of natural products is attributed to their molecular structures, thus to provide an explanation of the antioxidant activity of tested extracts, we used the ATR-IR technique to investigate the chemical composition of the extracts. The infrared spectroscopy method allows reliable, non-destructive distinction of the specific functional groups without further preparation requirements, and thus creating evidence of the species diversity. We report here for the first time the ATR-FTIR spectroscopic characterization of B. obscura outer and inner bark extracts. In general, all bands in the spectrum of the outer bark of *B. pendula* agree with literature reports on the bands that represent betulin, betulinic acid, and other triterpenes [33]. The FT-IR spectral pattern of the inner bark from *B. pendula* and *B. obscura* is rather similar, demonstrating the same main band positions and relative intensities. Hence, this confirms that the diversity of properties of both species resulting from differences in the structure of components of their bark. The outer bark is rich in pentacyclic triterpenoids, which include betulin and betulinic acid. The vibrational range of 1032–1006 cm^−1^ reflects the triterpene contribution and in theory, allows differentiation [22]. According to our data, it is challenging to distinguish between different triterpenes. Pure betulin and betulinic acid exhibit strong, distinguishable bands, but in the case of extracts, where the chemical composition of compounds is much greater, there is a broad band which makes it impossible to distinguish individual substances. The black birch outer bark spectrum possesses additional bands that are not attributable to either betulin or betulinic acid. These compounds may be responsible for additional antioxidant effects and may have other medical uses that require further elucidation.

Next, to confirm the presence of the main pentacyclic triterpenes in *B. obscura* bark extracts, we assessed the contents of betulin, betulinic acid, and lupeol, using high-performance liquid chromatography analysis (HPLC-PDA). Much to our surprise, it revealed considerable differences in betulin and lupeol levels in the inner bark extracts. Substantially higher contents were attributed to the extracts of *B. obscura* than to *B. pendula*, although the literature so-far denies the presence of betulin in *B. obscura* bark. In the outer bark, the results corresponded to the predictions, and both betulin and lupeol contents were higher in *B. pendula*. A similar concentration of betulinic acid was also found in all four extracts (Table 4). Thus, the results of our analysis contradict the literature description of *B. obscura* outer bark as entirely betulin-devoid. Because known literature sources attribute the difference in the bark color of *B. pendula* (as well as *B. alba,* etc.) and *B. obscura* to the absence of betulin in the latter, there must exist additional substances or as yet unknown mechanisms that impact this difference. Nevertheless, it is not true, as has been shown in our study, that *B. obscura* completely lacks betulin.

## 4. Materials and Methods

### 4.1. Preparation of the Bark Extracts from B. pendula and B. obscura

Plants were obtained courtesy of the Botanical Garden of The Maria Curie-Skłodowska University in Lublin (Lublin, Poland). The barks were collected in spring 2019. Plant preparations were washed with PBS solution and subjected to freeze-drying. Before that, the bark was frozen to −70 °C in glass containers intended for freeze-drying. A dryer by LABCONCO, model FreeZone 12, was used for the process, at a collector temperature of −50 °C. Freeze-drying was carried out at 30 °C, 0.09 mBar, for 48 h. After the procedure was completed, 50 g of dried, freeze-dried black and silver birch bark (outer and inner layers) were placed in a round-bottomed flask. A measure of 200 mL of ethanol was added, the resulting mixtures were heated under slight reflux for 4 h and then left overnight to cool down. Then the contents of the flasks were filtered through a funnel to obtain an extract from which the remaining excess alcohol was distilled. The next step was to add 20 mL of PBS to the dry matter of the extract. 

### 4.2. Cell Culture

The tests were performed on mouse L929 fibroblast cultures (cell line origin—mouse C3H/An connective tissue) from ATCC (Manassas, VA, USA). The cell line was tested for mycoplasma contamination with microbiological assays. The cultures were grown in 25 mL bottles under standard conditions (5% CO_2_ saturation, 37 °C, humidity 90%) with the addition of MEM (Minimum Essential Medium, Corning, New York, NY, USA) enriched with 5% fetal serum bovine (FBS, Pan-Biotech, Aidenbach, Germany) and antibiotics (100 U/mL penicillin, 100 μg/mL streptomycin, 0.25 μg/mL amphotericin B). Cells were passaged with 0.25% trypsin every third day or other, depending on the confluence. The cell density for further experiments was 2–4 × 10^4^ cells/mL. 

### 4.3. Methods for Assessing the Extracts Cytotoxicity

To access cytotoxicity of extracts two mechanism-independent assays were used. For this purpose, L929 cell lines were cultured for 24 h in 96-well plates. Then 20 µL of PBS-diluted extracts (2–100%) were added to the cells and incubated for 24 and 48 h. All absorbance measurements were made on a BioTek Epoch ELISA plate reader (BioTek Instruments, Winooski, VT, USA). The percentage of intact cells was calculated in relation to untreated cells.

#### 4.3.1. MTT Analysis

The principle of the assay is based on the ability of vital cells with intact mitochondrial membrane to reduce yellow, 3-(4,5-dimethyl-1,3-thiazol-2-yl)-2,5-diphenyl-2H-tetrazole bromide to purple formazan. The amount of colorful product is directly proportional to the metabolic activity of the cell [34]. 

After incubation with extracts, 5 mL MTT was added to the cells. Then, after 3 h, 100 mL of medium was withdrawn from each well and quenched with the same amount of DMSO. After 5 min shaking, absorbance at λ = 570 nm was read.

#### 4.3.2. Determination of Lactate Dehydrogenase (LDH)

The LDH assay determines the release of the enzyme that converts pyruvate to lactate in the presence of NADH. Lactate dehydrogenase is a cytoplasmic enzyme that is released to culture medium as a result of loss of cell membrane integrity, which can lead to cell death [34].

After incubation, 50 µL of the substrate, which was a mixture of NADH with pyruvate and diaforese, was added to the well. Plates were incubated for 30 min at room temperature. Then, 50 µL of acetic acid was added to stop the reaction. Absorbance was immediately measured at λ = 492 nm. 

### 4.4. Methods for Assessing the Antioxidant Mechanisms of Extracts

L929 cells were treated with acetaldehyde (175 μM) as an oxidative stress inducer (24 h incubation). Then, based on cytotoxicity studies, two concentrations of extracts below IC_50_, namely 10%, and 25% were selected as potential antioxidative stress treatment and incubated for another 24 and 48 h. Subsequently, hydrogen peroxide (H_2_O_2_), superoxide anion radical (O_2_^•−^) and malonic dialdehyde (MDA) concentration was determined for elucidating the antioxidant mechanism.

#### 4.4.1. Determination of Hydrogen Peroxide (H_2_O_2_)

The test is based on the reaction of horseradish peroxidase, which breaks down the resulting hydrogen peroxide in the presence of phenol red as a chromogen (change from red to yellow) [35].

The cells were washed twice with Hanks Balanced Salt Solution (HBSS). After that, 100 µL of the mixture: HBSS solution, phenol red (Sigma-Aldrich, St. Louis, MO, USA)—final concentration of 0.56 mM, and HRPO (Serva, Heidelberg, Germany—final concentration of 20 U/mL) were added to each well. In addition, 10 µL of 1M NaOH was added to each well to maintain the correct pH for phenol red, and the plate was incubated for one hour at 37 °C. Then, all cells were again washed twice with HBSS solution and intracellular hydrogen peroxide production was measured at 600 nm. The results were presented as H_2_O_2_ nanomoles per 10^6^ cells produced in 60 min, based on the coextrusion coefficient for phenol red (ΔE600) of 19.8 × 10^3^ M^−1^ cm^−1^ (according to the manufacturer’s protocol).

#### 4.4.2. Determination of Superoxide Anion Radical (O_2_^•−^)

The cells were washed with Hanks Balanced Salt Solution (HBSS). Then a mixture of HBSS solution, cytochrome c solution (final concentration 75 μM), with SOD (final concentration 60 U/mL) or without SOD was added to some of the cells. Cells were incubated for 60 min at 37°C after which absorbance was read at 550 nm. The absorbance of the samples was converted into O_2_^•−^ nanomoles based on the cytochrome C co-extinction coefficient: ΔE550 = 21 × 10^3^ M^−1^ cm^−1^ [36]. The results were presented as O_2_^•−^ nanomoles per 10^6^ cells produced by the cells in 60 min.

#### 4.4.3. MDA Assay

The determination of the malonic dialdehyde (MDA) as an indicator of lipid peroxidation was performed by colorimetry using the Bioxytech LPO-586 reagent kit (OxisResearch, Portland, OR, USA), according to the procedure attached to the assay kit. The level of malonic dialdehyde was measured at λ = 586 nm. 

### 4.5. Infrared Spectroscopy

For recording infrared (IR) spectra in the 4000–400 cm^−1^ range, a Thermo Scientific Nicolet 6700 FTIR spectrophotometer (Madison, WI, USA) equipped with ATR (Attenuated Total Reflectance) with a diamond crystal, controlled by Omnic 8.2 software was used. The background spectrum was recorded at the beginning of the measurements. The number of scans was 32 and the spectral resolution was 4 cm^−1^. The results were presented in the form of transmittance. The spectral data have been processed using SpectraGryph 1.2 and Origin 8.0 software.

### 4.6. High Performance Liquid Chromatography with Photodiode Array (HPLC-PDA)

Presence and quantification of specific pentacyclic triterpenes in dry extracts of birch extracts, betulinic acid, betulin, lupeol were assessed using previously published methods [37,38]. For lupeol determination RP18e Chromolith 100 column (10 cm × 2.0 mm i.d. Merck, Darmstadt, Germany) with ACN/H_2_O (95:5 *v*/*v*) as mobile phase at flow rate of 2 mL/min. and column temperature 25 °C was used. While RP18e LiChrospher 100 column (25 cm × 4.0 mm i.d. 5 µm, Merck) with mobile phase ACN/H_2_O/H_3_PO_4_ (75:25:0.5 *v*/*v*/*v*) at flow rate of 1 mL/min. and column temperature 10 °C was applied. Wavelength λ = 200 nm was used for quantitative analysis.

### 4.7. Statistical Analysis 

The obtained experimental results were subjected to statistical analysis using the GraphPad Prism 8. Measures were analyzed using a one-way analysis of variance (ANOVA) with Dunnett’s post-test. The results were expressed as mean standard deviation (SD) of three to four independent repetitions. IC_50_ was calculated by nonlinear, four parameters regression analysis.

## 5. Conclusions

In conclusion, using an in vitro oxidative stress model our studies showed the strong antioxidative potential of silver and black birch bark extract by reducing the production of H_2_O_2_, O_2_^•−^ and MDA. Chemical profiling revealed the similar composition of birch bark and an equally high content of triterpenes in black birch as in white birch. Furthermore, this study creates a solid basis for further hypotheses and extended research. To confirm the antioxidant properties of a rather unknown species, which *B. obscura* undoubtedly is, more advanced and antioxidation tests, techniques (HPLC-TBARS) [39], models (in vivo), appear to be the natural continuation of the study. To elucidate which compounds may contribute to the freshly discovered properties of *B. obscura*, the chemical composition of its bark should be comprehensively analyzed in the future.

## Figures and Tables

**Table 1:** Vibrational frequencies (cm^−1^) with assignments of *B. pendula* and *B. obscura* outer bark extracts and reference standards of betulin and betulinic acid.

*Betula pendula* o.b.	*Betula obscura* o.b.	Betulin	Betulinic Acid	Assignments
3342 br	-	3459–3363 m, br	3443 w	ν(OH)
2969 sh	-	2968 sh	-	ν_s_, ν_as_ (CH_3_, CH_2_, CH)
2929 vs	2928 m	2929 s	2940 s	
2868 s	2852 m	2867 m	2868 s	
1737 br, 1681 sh	1737–1717 s, br	1735 w, 1708 sh	1737 sh, 1684 vs	ν(C=O)
1612 w	1606 m	-	-	-
1515 w	1515 m	-	-	-
1448 s	1447 m	1451 m	1448 m	δ(CH_3_) + δ(CH_2_)
1365 s	1374 m	1374 m	1375 m	δ(CH_3_) + δ(CH_2_)
-	1259 m	-	-	-
1229 m	1228 m	-	1231 s	δ(OH) + τ(CH_2_) + δ(CH)
1217 m	1217 m	-	1218 sh	-
1105	1102 w	1105 m	1108 m	-
1081	1070 w	1083 m	-	-
1028 vs, br	1028 s	1035 m, 1008 vs	1043 m, 1035 sh, 1010 m	ν(C-O) + δ(CH) + ρ(CH_3_, CH_2_)
983 sh	982 sh	984 m	983 w	-
880 m	880 m	875 vs	884 s	ω(H-C-H)
-	800 vs, br	-	791 w	

Abbreviations: i.b.: inner bark, o.b.: outer bark, br: broad, m: medium, sh: shoulder, s: strong, v: very, w: weak, ν: stretching, δ: bending, τ: torsion, ρ: rocking, ω: wagging.

**Table 2:** Content of triterpenes (mg/g dry extracts ± SD) (*n* = 3).

Bark Extract		Betulinic Acid	Betulin	Lupeol
*Outer*	*B. pendula*	97.42 ± 3.91	295.93 ± 3.94	40.04 ± 4.91
	*B. obscura*	49.89 ± 0.74	154.86 ± 1.01	52.42 ± 0.02
*Inner*	*B. pendula*	56.89 ± 2.43	417.49 ± 2.02	78.64 ± 11.44
	*B. obscura*	48.77 ± 3.50	424.45 ± 5.87	127.36 ± 7.13

**Table 3:** Table summarizing percentage of cell viability after the exposition to the tested extracts, acquired by MTT assay after 24 and 48 h incubation. One hundred percent of viable cells correspond to the values obtained in the control culture (0% extracts). Data presented as the means ± SD.

Conc./Extract	*B. pendula* i.b.		*B. pendula* o.b.		*B. obscura* i.b.		*B. obscura* o.b.	
	24 h	48 h	24 h	48 h	24 h	48 h	24 h	48 h
100%	36.6 ± 2.8 ****	35.9 ± 1.8 ^^^^^^	35.9 ± 2.5 ****	32.7 ± 3.3 ^^^^^^	35.3 ± 3.7 ****	29.4 ± 1.1 ^^^^^^	32.8 ± 3.4 ****	27.8 ± 0.2 ^^^^^^
75%	36.2 ± 3.8 ****	31.7 ± 4.4 ^^^^^^	41.0 ± 1.0 ****	33.5 ± 3.1 ^^^^^^	35.7 ± 3.4 ****	34.0 ± 3.1 ^^^^^^	36.1 ± 2.0 ****	31.1 ± 2.0 ^^^^^^
50%	50.2 ± 1.5 ****	39.5 ± 1.3 ^^^^^^	51.3 ± 7.7 ****	38.3 ± 9.2 ^^^^^^	40.3 ± 0.5 ****	34.5 ± 1.6 ^^^^^^	42.3 ± 5.5 ****	39 ± 2.7 ^^^^^^
25%	60.5 ± 10.4 ***	60.6 ± 1.8 ^^^^^^	57.6 ± 2.8 ****	61.5 ± 0.9 ^^^^^^	64.1 ± 4.7 ****	56.2 ± 6.8 ^^^^^^	60.3 ± 4.2 ****	53.1 ± 5.8 ^^^^^^
10%	77 ± 8.2 *	63.2 ± 7.8 ^^^^^^	63 ± 4.5 ****	64.5 ± 5.3 ^^^^^^	68.8 ± 1.1 ****	60.2 ± 3.6 ^^^^^^	64.3 ± 4.7 ****	61.5 ± 6.1 ^^^^^^
5%	83.6 ± 8.5	83.8 ± 4.8	95.9 ± 3. 9	80.3 ± 4.5 ^^^^	91.9 ± 5.8	81.4 ± 5.8 ^^^^	91.9 ± 2.8	76.8 ± 1.7 ^^^^^^
2%	102.6 ± 5.6	102.1 ± 8.7	100.2 ± 7.7	94.4 ± 3.5	104.0 ± 4.8	101.0 ± 7.7	99 ± 7.4	96 ± 5
IC_50_ [%]	43	33	43	31	39	28	36	26

* *p* < 0.05, *** *p* < 0.001, **** *p* < 0.0001 vs. 24 h treated negative control group, ^^^^ *p* < 0.01, ^^^^^^ *p* < 0.0001 vs. 48 h treated negative control group; Dunnett’s test. Abbreviations: i.b., inner bark; o.b., outer bark.

**Table 4:** Table summarizing percentage of LDH release after the exposition to the tested extracts, acquired by LDH assay after 24 and 48 h incubation. The value of 100% corresponds to the highest readings. Data presented as the means ± SD.

Conc./Extract	*B. pendula* i.b.		*B. pendula* o.b.		*B. obscura* i.b.		*B. obscura* o.b.	
	24 h	48 h	24 h	48 h	24 h	48 h	24 h	48 h
100%	97.1 ± 5.7 ****	94.6 ± 8.6 ^^^^^^	91.0 ± 5.2 ****	99.5 ±15.2 ^^^^^^	98.9 ± 4.4 ****	103.5 ± 6.9 ^^^^^^	108.3 ± 8.1 ****	100.1 ± 6.5 ^^^^^^
75%	74.7 ± 7.2 ****	82.2 ± 2.4 ^^^^^^	69.8 ± 4.7 ****	77.7 ± 6.7 ^^^^^^	85.4 ± 4.6 ****	84.3 ± 13.7 ^^^^^^	83.4 ± 5.2 ****	79.6 ± 2.7 ^^^^^^
50%	40.5 ± 4.5 ****	46.9 ± 5.5 ^^^^^^	39.2 ± 4.7 ****	55.8 ± 7.8 ^^^^^^	60.9 ± 7.0 ****	63.5 ± 3.7 ^^^^^^	59.3 ± 2.7 ***	62.3 ± 3.8 ^^^^^^
25%	28.3 ± 2.6 ***	27.3 ± 5.0	24.6 ± 2.8 ***	25.3 ± 2.2	31.3 ± 2.2 ****	42.0 ± 6.8 ^^^^^	35.9 ± 1.6 *	34.7 ± 3.0 ^^^^^^
10%	18.2 ± 2.1	17.3 ± 9.1	13.3 ± 1.6	11.3 ± 1.3	21.4 ± 4.5 **	23.6 ± 3.9	23.4 ± 5.3	19.4 ± 6.0
5%	12.7 ± 0.6	9.8 ± 2.8	8.1 ± 0.9	7.3 ± 2.8	11.6 ± 0.9	13.5 ± 1.4	11.0 ± 0.9	8.4 ± 0.9
2%	10.7 ± 0.8	9.7 ± 4.4	8.1 ± 1.0	6.9 ± 1.0	7.4 ± 4.0	8.7 ± 1.7	21.4 ± 20.4	7.7 ± 1.9
IC_50_ [%]	61	54	61	48	44	39	45	42

* *p* < 0.05, ** *p* < 0.01, *** *p* < 0.001, **** *p* < 0.0001 vs. 24 h treated negative control group, ^^^^^ *p* < 0.001, ^^^^^^ *p* < 0.0001 vs. 48 h treated negative control group; Dunnett’s test. Abbreviations: i.b., inner bark; o.b., outer bark.

## Data Availability

The data presented in this study are available on request from the corresponding author.

## Supplementary Materials

The following are available online, Figure S1: FT-IR spectrum of *B. pendula* outer bark extract, Figure S2: FT-IR spectrum of *B. obscura* outer bark extract, Figure S3: FT-IR spectrum of *B. pendula* inner bark extract, Figure S4: FT-IR spectrum of *B. obscura* inner bark extract, Figure S5: FT-IR spectrum of betulin standard, Figure S6: FT-IR spectrum of betulinic acid standard.

## Sample Availability

Samples of the compounds are not available from authors.
