# Supplementary figures and images for: Evaluation of Antioxidative Mechanisms In Vitro and Triterpenes Composition of Extracts from Silver Birch (Betula pendula Roth) and Black Birch (Betula obscura Kotula) Barks by FT-IR and HPLC-PDA
Source: Molecules. 2021 Jul 30;26(15):4633. doi: 10.3390/molecules26154633 (PMC8347892; doi:10.3390/molecules26154633)

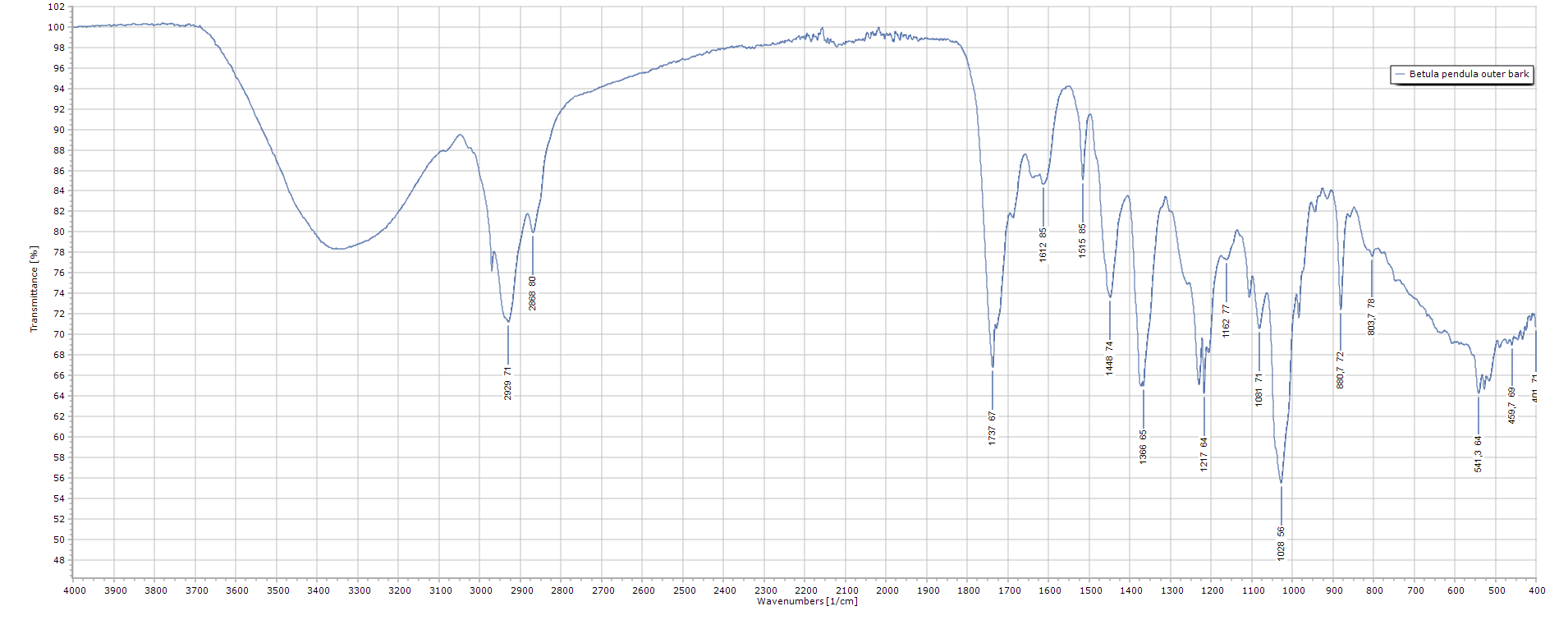

Supplement: Supplementary file 1 [file molecules-26-04633-s001.zip › supplementary/Figure S1.png]

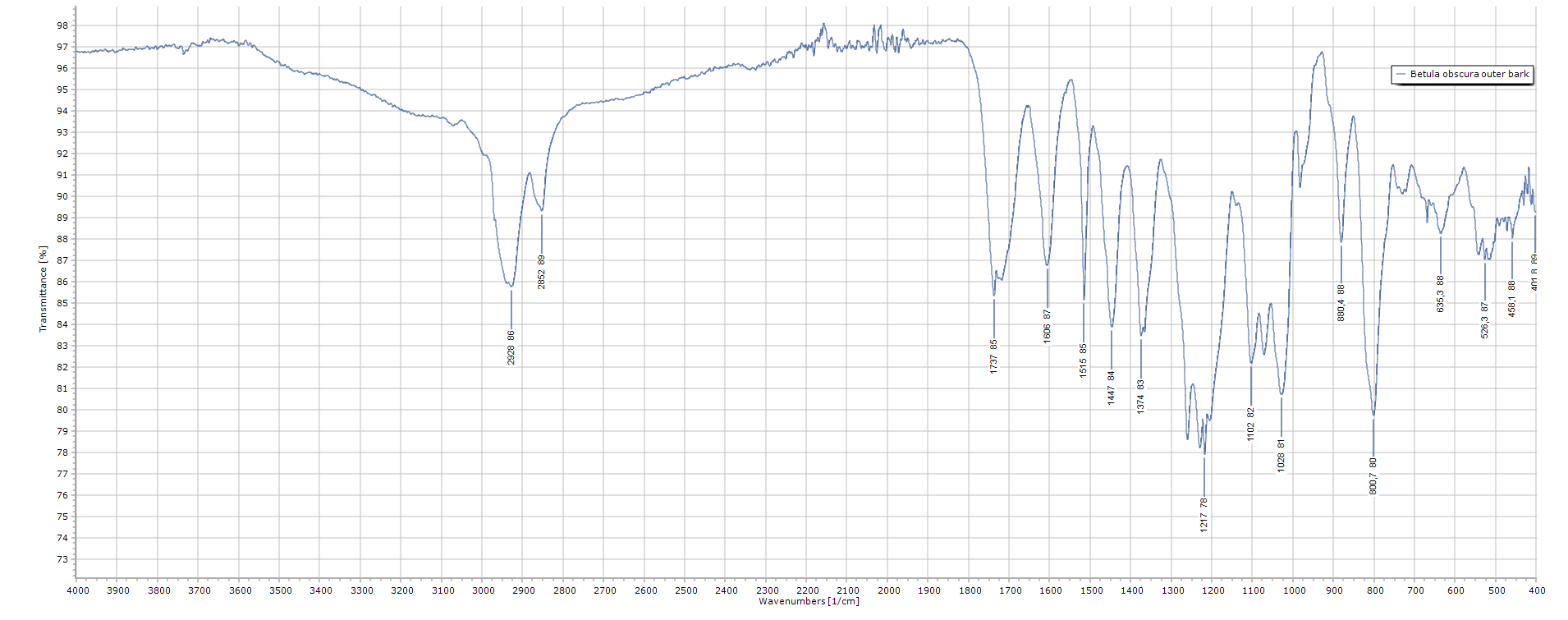

Supplement: Supplementary file 1 [file molecules-26-04633-s001.zip › supplementary/Figure S2.png]

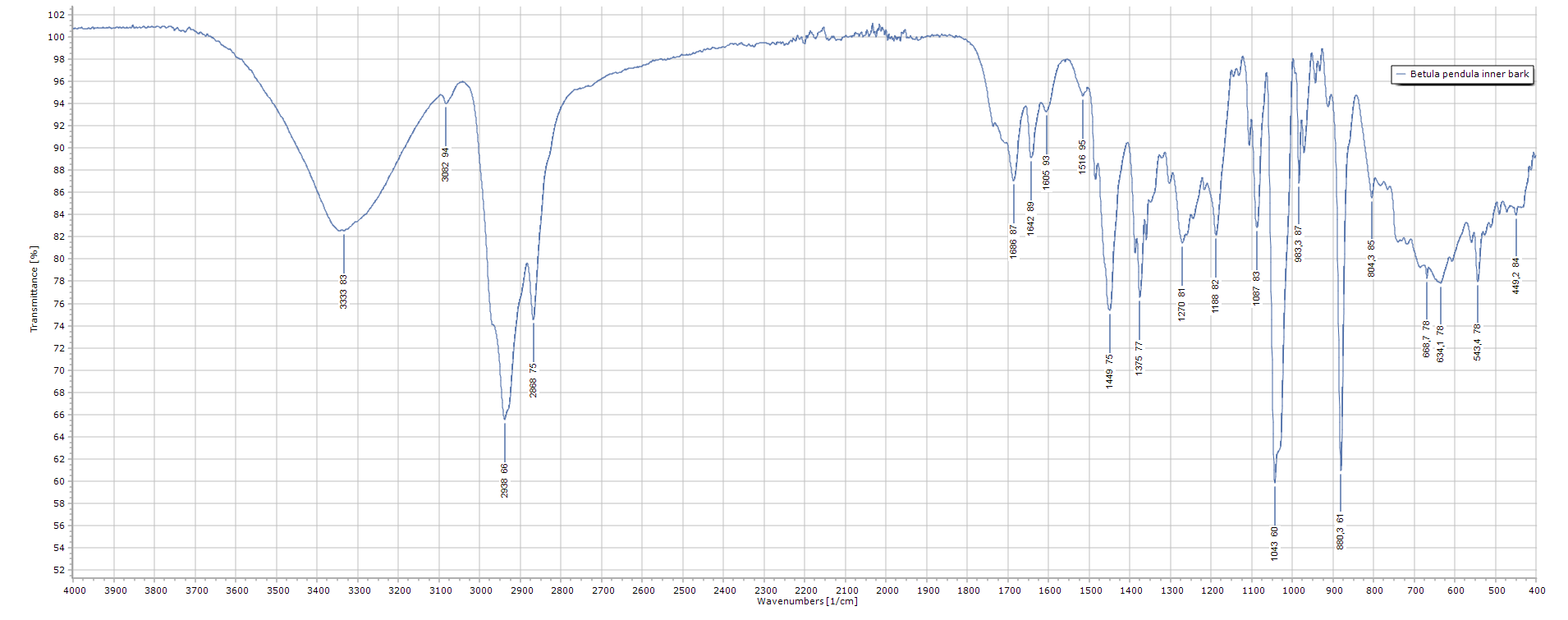

Supplement: Supplementary file 1 [file molecules-26-04633-s001.zip › supplementary/Figure S3.png]

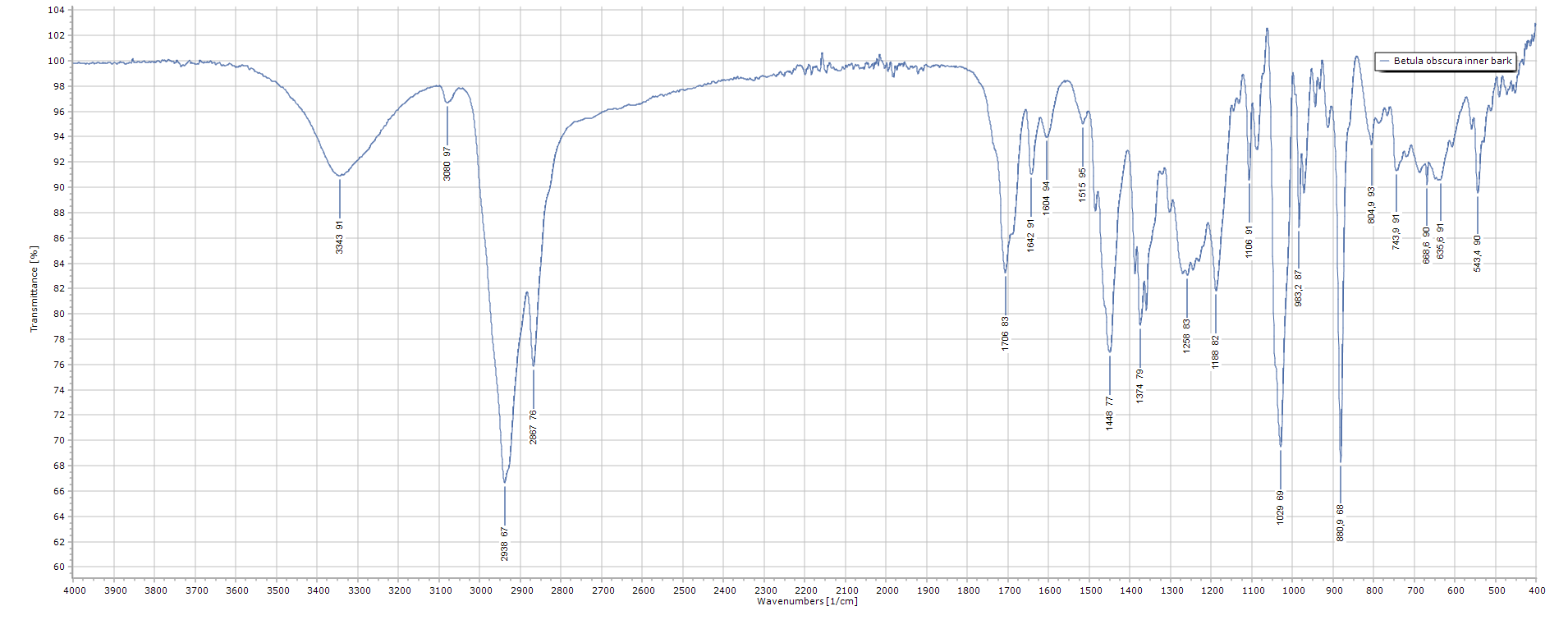

Supplement: Supplementary file 1 [file molecules-26-04633-s001.zip › supplementary/Figure S4.png]

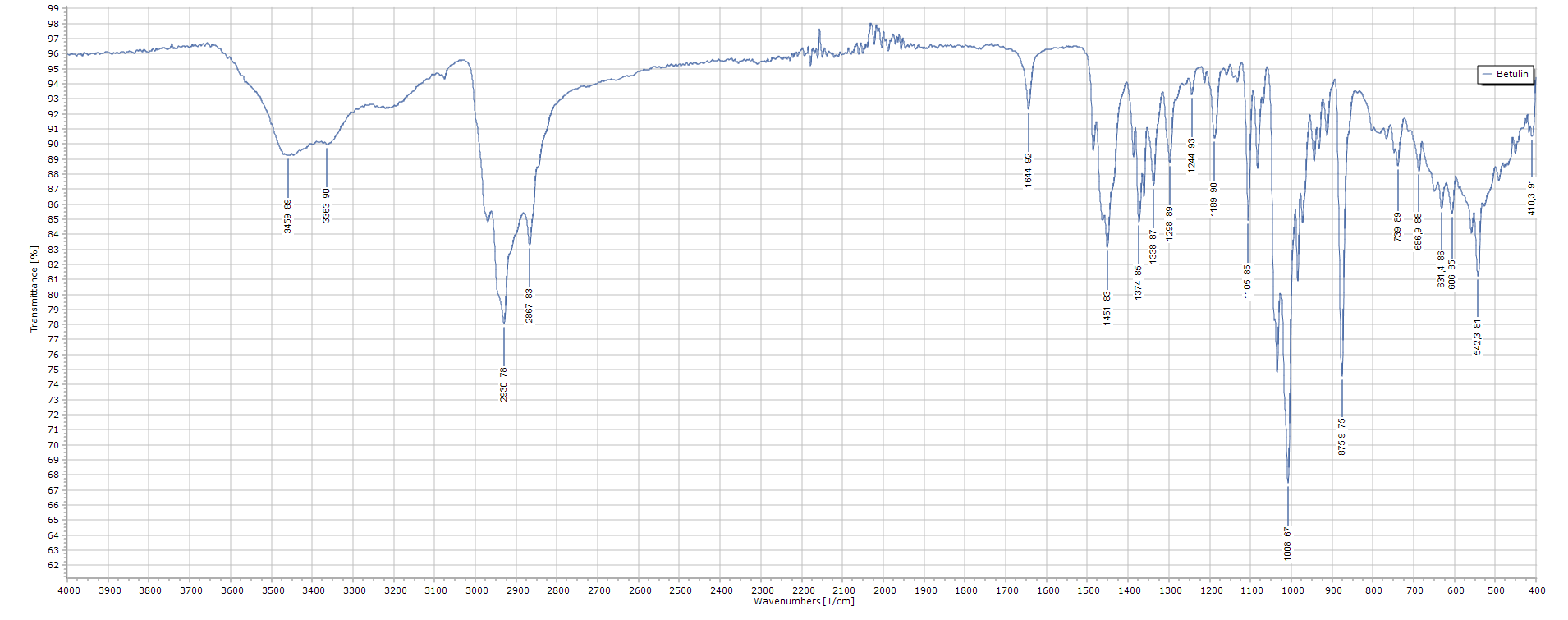

Supplement: Supplementary file 1 [file molecules-26-04633-s001.zip › supplementary/Figure S5.png]

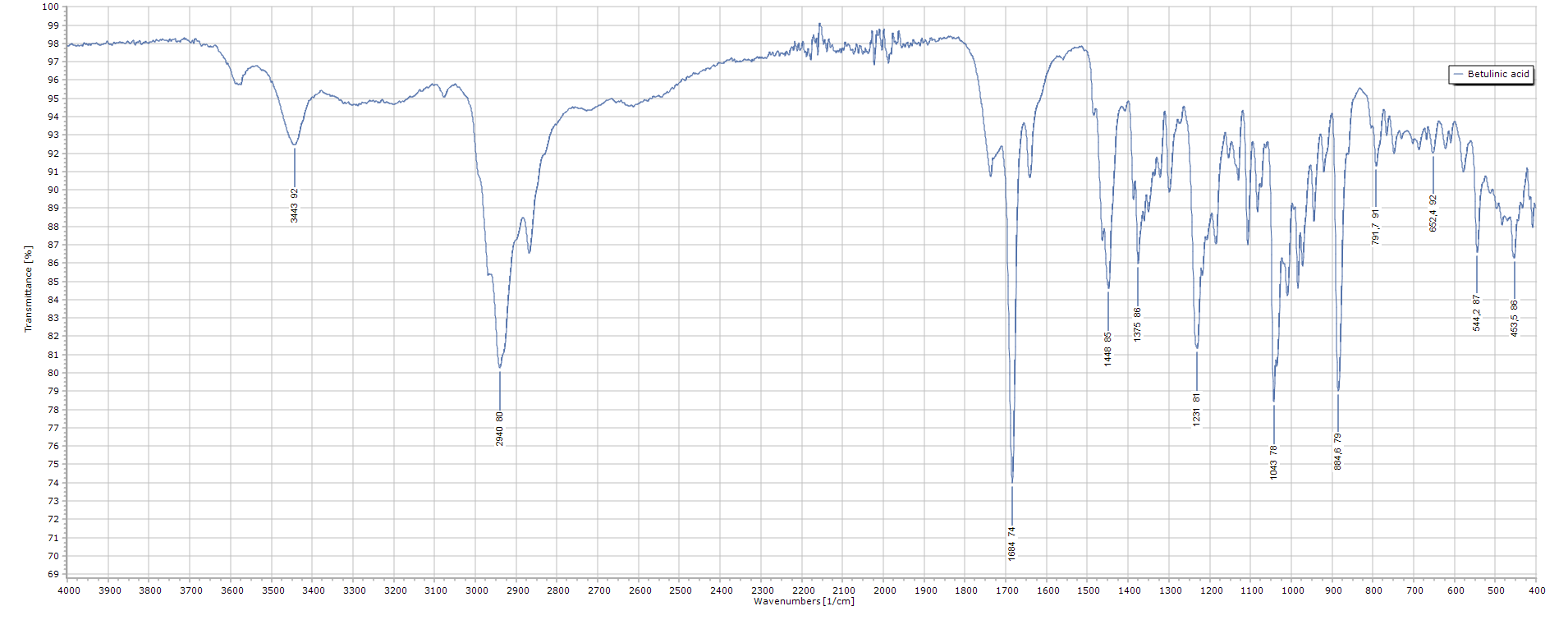

Supplement: Supplementary file 1 [file molecules-26-04633-s001.zip › supplementary/Figure S6.png]
